# ADAM15 gene structure and differential alternative exon use in human tissues

**DOI:** 10.1186/1471-2199-8-90

**Published:** 2007-10-15

**Authors:** Iivari Kleino, Rebekka M Ortiz, Ari-Pekka J Huovila

**Affiliations:** 1 Institute of Medical Technology, University of Tampere, Tampere, Finland; 2 Department of Pathology, Tampere University Hospital, Tampere, Finland; 3 Haartman Institute, Dept. Virology, University of Helsinki, Helsinki, Finland

## Abstract

**Background:**

ADAM15 is a metalloprotease-disintegrin implicated in ectodomain shedding and cell adhesion. Aberrant *ADAM15 *expression has been associated with human cancer and other disorders. We have previously shown that the alternative splicing of *ADAM15 *transcripts is mis-regulated in cancer cells. To gain a better understanding of ADAM15 regulation, its genomic organization and regulatory elements as well as the alternative exon use in human tissues were characterized.

**Results:**

Human *ADAM15*, flanked by the FLJ32785/DCST1 and *ephrin-A4 *genes, spans 11.4 kb from the translation initiation codon to the polyadenylation signal, being the shortest multiple-exon *ADAM *gene. The gene contains 23 exons varying from 63 to 316 bp and 22 introns from 79 to 1283 bp. The gene appeared to have several transcription start sites and their location suggested the promoter location within a CpG island proximal to the translation start. Reporter expression experiments confirmed the location of functional GC-rich, TATAless and CAATless promoter, with the most critical transcription-supporting elements located -266 to -23 bp relative to the translation start. Normal human tissues showed different complex patterns of at least 13 different *ADAM15 *splice variants arising from the alternative use of the cytosolic-encoding exons 19, 20a/b, and 21a/b. The deduced ADAM15 protein isoforms have different combinations of cytosolic regulatory protein interaction motifs.

**Conclusion:**

Characterization of human *ADAM15 *gene and identification of elements involved in the regulation of transcription and alternative splicing provide important clues for elucidation of physiological and pathological roles of ADAM15. The present results also show that the alternative exon use is a physiological post-transcriptional mechanism regulating *ADAM15 *expression in human tissues.

## Background

*ADAM15 *is a widely expressed member of the *ADAM *(adisintegrin and metalloprotease) gene family encoding transmembrane metalloproteinase-disintegrins [1]. The characteristic domain structure of ADAM proteins consists of N-terminal signal sequence and prodomain, which typically are proteolytically excised during biosynthetic maturation, followed by metalloproteinase, disintegrin, and cysteine-rich domains, a transmembrane segment, and a cytosolic tail. Despite the presence of the conserved metalloproteinase domain, not all ADAMs are active proteinases; roughly half of the over 30 ADAM members do not contain all catalytically critical residues. Human ADAM15 is an active metalloproteinase and can bind to several integrins [2].

ADAMs have been implicated in numerous physiological and pathological processes through their proteolytic or adhesive activities, including ectodomain shedding and cell-cell interactions [2-5]. ADAM15 has been implicated in activation of growth factors through ectodomain shedding and in cell-cell interactions, but specific physiological roles are not known. *ADAM15 *gene knockout in mice did not result in obvious developmental or pathological phenotype [6,7]. However, strongly decreased angiogenesis in the retinopathy of prematurity model in *adam15*^-/- ^mice indicated a critical role in pathological neovascularization [6], corroborated by the studies implicating ADAM15 in regulation of vasculogenesis [8-11].

Altered *ADAM15 *expression has been associated with human diseases, including cancers, cardiac disease, atherosclerosis, and arthritis [9,12-18]. In cancers, ADAM15 has been implicated in tumor growth, angiogenesis, and metastasis [6,8,18]. In addition to the transcriptional regulation of *ADAM15 *expression, alternative splicing of *ADAM15 *transcripts has been shown to be mis-regulated in human cancer cells [19]. Although *ADAM15 *splice-variants have been found from mouse and human cell lines [19-22], no information has been available about the alternative *ADAM15 *splicing in normal tissues.

To gain better understanding of ADAM15 regulation, the present study provides a detailed structural and functional characterization of human *ADAM15 *gene as well as its expression and alternative exon use in normal human tissues.

## Results

### Human *ADAM15 *gene structure

**Figure 1:**
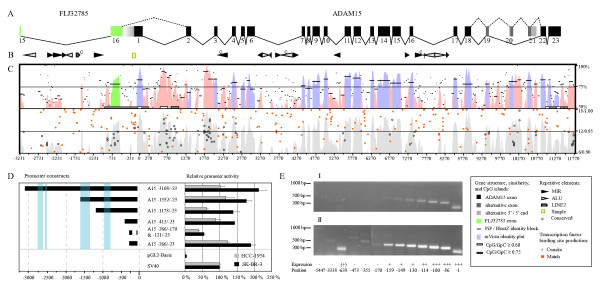
Human *ADAM15 *gene structure, phylogenetic footprinting, promoter and transcription start site analysis. The panels (A), (B), (C), and the left part of the panel (D) are in register respective to the nucleotide numbering shown under the panel (C). The symbol key for the panels (A) – (C) is shown at bottom right. (A) *ADAM15 *exon-intron structure is depicted with black and grey boxes representing the constitutively and alternatively used exons, respectively. Exon numbering is indicated under the boxes. The alternative exon 20a/b and 21a/b 5'- and 3'-ends are indicated with pale grey. The green boxes represent the last two exons (exons 15 and 16) of the upstream gene FLJ32785. The spaces separating the exons are proportional to the intron lengths. The solid lines connecting the *ADAM15 *exons represent the most frequently observed splice pattern corresponding to the *ADAM15 *mRNA variant 2. The dashed lines connecting the exons 18–22 represent the alternative splicing producing the *ADAM15 *variant with the longest open reading frame (variant 6). The dashed line between the FLJ32785 exon 16 and the *ADAM15 *exon 2 depicts the observed fusion splicing between the two genes. The panel (B) shows the repetitive element locations. Conservation of repetitive elements between human and mouse is indicated by the letter c. (C) The upper part shows the sequence-similarity between the mouse and human genes, indicated by the identity percentage on the vertical axis; the mVista sequence-identity plot is shown with pink shading in the intronic regions and with blue or green shading in the exonic regions. The PiP-maker identity blocks are shown with black horizontal lines. CpG islands are indicated with white and grey boxes (see the symbol key). The lower part of the panel C shows the locations of predicted transcription factor binding sequences with the relative strength of prediction on the vertical axis; the Match-program was used to predict the conventional binding motifs (solid orange squares) and the Consite for phylogenetically conserved binding motifs (open circles); predictions with scores ranging from 0.90 to 1.00 are shown for Match and from 5 to 15 for Consite. The location relative to the *ADAM15 *translation start ATG is indicated by the nucleotide numbering under the panel. (D) *ADAM15 *promoter analysis with luciferase-reporter expression. The left side of the panel D shows the *ADAM15 *upstream regions examined in the luciferase reporter experiments. The light blue shading indicates the conserved regions outside of the repetitive elements. The nucleotide location relative to *ADAM15 *translation start ATG, in register with the panel (C), is shown under the panel. The right side of the panel (D) shows the relative promoter activity of the reporter constructs in comparison to the pGL2-Basic control plasmid and to the SV40 promoter. The black and grey bars indicate the promoter activity measured in SK-BR-3 and HCC-1954 cells, respectively. (E) The transcription start site analysis using RT-PCR described in the Materials and Methods. The position of the upstream primer relative to *ADAM15 *translation start ATG in each PCR is indicated under the electrophoresis lanes. The upper gel shows the products of 30 PCR cycles and the lower gel those of 35 PCR-cycles.

PAC-clone 240C8 containing the human *ADAM15 *gene was previously identified and shown to hybridize with a single chromosomal locus at 1q21.3 [23]. Sequencing verified that 240C8 includes the entire *ADAM15 *gene (Fig. 1A). The annotated gene sequence was submitted to GenBank [GenBank: AF314227].

*ADAM15 *gene is flanked by the FLJ32785/DCST1 and *ephrin-A4 *(EFNA4) genes. FLJ32785 (NCBI Entrez GeneID 149095) polyadenylation signal is located 477 bp upstream from *ADAM15 *translation start codon, and *ephrin-A4 *(GeneID 1945) translation start 1069 bp downstream from ADAM15 polyadenylation signal. In this report, if not mentioned otherwise, the nucleotide numbering is relative to the adenine of the *ADAM15 *translation start codon.

Human *ADAM15 *gene spans 11367 bp from the translation start to the polyadenylation signal, contains 23 exons varying from 63 to 316 bp, and 22 introns varying from 79 to 1283 bp. (Fig. 1A). *ADAM15 *is the shortest multiple-exon *ADAM *gene (comparison data not shown). The exons 19–21 are used alternatively in human tissues (Fig. 1A).

All *ADAM15 *introns conform the GT-AG splicing rule (Additional file 1). While the *ADAM15 *introns in general contain good putative branch sites, the branch sites in the introns 3, 6, 10, and 19 scored lower than average branch sites in the human genome. Although the Branch-Site Anlyzer failed to predict the branch site for intron 1, a good consensus sequence was found at appropriate location. The putative branch site sequences are shown in Additional file 1.

The intron/exon structure of the human, mouse, and rat *ADAM15 *genes is almost identical. The human exon 2, the mouse exon 6, and the rat exon 20 show an internal 3 bp deletion respective to the others. Another difference is that the 3'-splice site (3'ss) of the human intron 6 is located three nucleotides downstream from that of the rodent genes, causing the human exon 7 to be 3 bp shorter. The ag corresponding to the 3'ss of the rodent intron 6 is also present in the human sequence (Additional file 1) and hence the human intron 6 acceptor contains a tagtag-sequence constituting a candidate NAGNAG tandem acceptor [24] which could enable 3 bp variation at the exon start. Use of the proximal acceptor would lead to an inframe stop codon (TAG) at the exon 7 start. However, transcripts with the exon 7 stop form have not been detected.

The exons 20 and 21 have alternative 5'- or 3'-ends, respectively (Additional file 1). The exon 20 forms, the shorter referred as 20a and the longer as 20b, are a consequence of a NAGNAG tandem acceptor (Additional file 1). The alternative exon 21 forms share the 5' splice site (5'ss) and the longer referred as 21b has a 3' splice site (3'ss) 142 bp further downstream from that of 21a (Additional file 1).

The *ADAM15 *gene contains four separate CpG-islands. The longest CpG-island extends from the last FLJ32785 intron to the first *ADAM15 *intron (Fig. 1C). Two shorter islands are located within *ADAM15 *intron 1, and the fourth island encompass region from *ADAM15 *exon 22 past the polyadenylation site (Fig. 1C).

### Human ADAM15 gene contains several transcription start sites and a TATAless promoter

#### Evidence for several transcription start sites

5'RACE experiments indicated the presence of several 5'-ends upstream of *ADAM15 *exon 2, differing in length and sequence (data not shown). However, an impractical number of 5'RACE products would have had to be cloned and sequenced for unambiguous results, due to alternative sequence in similar-sized 5'-ends. For the same reason, primer extension from exon 2 would have produced equivocal results. Therefore the PCR approach described in the Methods was used to analyse the 5' end of *ADAM15 *transcripts.

The gradual decrease of product levels in consecutive PCRs of 5'-UTR indicated that human *ADAM15 *transcription starts at several sites at different levels (Fig. 1E). While most *ADAM15 *transcriptions started at around 110 bp upstream from the translation start, transcripts starting from up to -159 bp were detected with 30 PCR cycles (Fig. 1E).

**Figure 2:**
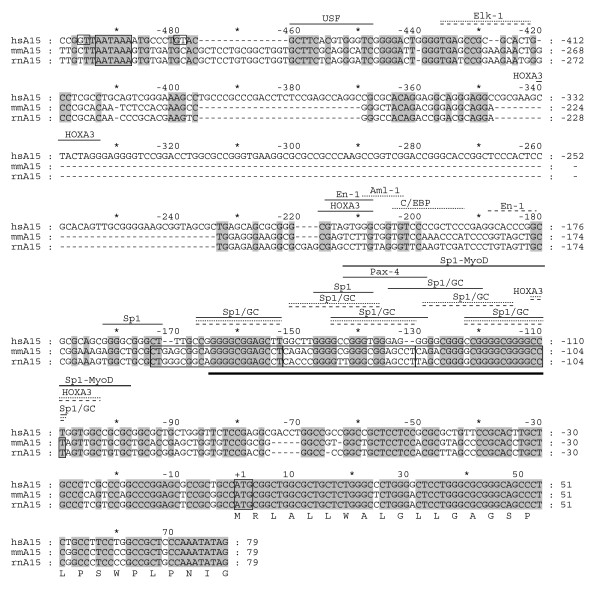
Comparative transcription factor binding site analysis of *ADAM15 *promoter. The human *ADAM15 *promoter and the first exon nucleotide sequences were aligned with corresponding mouse and rat sequences (hsA15, mmA15, and rnA15, respectively). The numbering above the sequences indicates position respective to the adenine in the translation start codon. The amino acid translation is shown for the first exon of human *ADAM15*. The predicted high-score transcription factor recognition motifs are indicated above the sequence (solid line, human; dotted line, mouse; dashed line, rat). The three conserved 22 bp repeated elements, the translation start ATG, and the polyadenylation signal of the upstream gene FLJ32785 are boxed. The boxed GTs indicate the detected splice donor sites in the last FLJ32785 exon used in splicing of human FLJ32785 to ADAM15. The thick line below the aligned sequences indicates the region containing the detected transcription start sites of the human *ADAM15 *gene.

The 5'RACE results also indicated fusion transcription, joining the upstream-neighbour FLJ32785 and *ADAM15*. This was confirmed using the PCR approach. Transcripts were amplified with a common downstream primer at *ADAM15 *exon 2 and various upstream primers at FLJ32785 exons 15 and 10. This indicated that fusion transcripts start at least within FLJ32785 exon 10, possibly containing FLJ32785 fused to *ADAM15*. However, the fusion transcripts were detected only after 35 PCR cycles, indicating their scarcity in the studied cells (Fig. 1E). 5'RACE product and GenBank EST sequences indicate that both GTs flanking the FLJ32785 polyadenylation signal were used as donors in splicing to *ADAM15 *exon 2 (Fig. 2).

The upstream primers at the FLJ32785 intron 15 failed to amplify PCR-products, suggesting that the intron 15 is excluded normally from fusion transcripts. *ADAM15 *transcripts with FLJ32785 intron 15 sequence at 5' end were not detected, indicating that no alternative *ADAM15 *transcription start sites are present in the FLJ32785 intron 15. Hence, the transcripts encoding ADAM15 protein start at -159 through -110 region. Given this and the location within a CpG island, this region was examined for promoter activity.

#### ADAM15 promoter induced expression in luciferase reporter assay

Reporter expression analysis confirmed that the candidate promoter region contained a functional promoter. The region -266 through -23 increased the reporter activity to 1.2- and 1.9-fold in SK-BR-3 and HCC-1954 cells, respectively, as compared to the control SV40-promoter (Fig. 1D).

Deletion of the nucleotides -171 to -122 resulted in the reduction of the promoter activity to a third of the original (Fig. 1D). This weakened mutant promoter lacked five of the six Sp1 recognition motifs and the Pax-4 site, containing only an En-1, a HoxA3, and a composite Sp1/MyoD recognition motif (Fig. 2).

The upstream segment -415 to -267 showed a weak repressive effect, since its addition to the -266 through -23 construct reduced the promoter activity (Fig. 1D). However, the corresponding sequence is largely absent from the mouse and rat *adam15 *genes (Fig. 2), and the only high score vertebrate transcription factor (TF) binding motif within this region is for HOXA3 (Fig. 2). While the reporter construct extension to the base pair 1178 did not alter the reduced promoting efficiency, the further extension to 1582 restored the reported expression to the original level (Fig. 1D). This suggests positive regulatory activity for the region 1582 to 1179. Extension of the reporter construct beyond 3000 did not significantly affect the promoter activity. The effect of the FLJ32785 intron 15 to *ADAM15 *promoter activity was minimal at most, consistent with the lack of conservation in the region. Altogether, the most critical *ADAM15 *transcription-supporting elements are located within the region 266 to 23.

#### Transcription factor binding motifs in the core promoter

The several transcription start sites 159 to 110 bp upstream from *ADAM15 *translation start indicated the location of a core promoter (Fig. 1C). The presence of several conserved TF recognition motifs in the region is consistent with the transcription start sites corroborating the core promoter location. The promoter region showed some conservation in automated sequence alignment (Fig. 1C). However, manual optimization increased the conservation significantly (Fig. 2).

The high-score vertebrate TF binding motifs found in human *ADAM15 *promoter sequence and in the corresponding mouse and rat sequences are shown in Figure 2. The putative *adam15 *promoter lacks the TATA and CAAT boxes also in rodents, and contains conserved binding sequences for five Sp1/GC-box recognition sites in all three species. In addition, the human sequence contains a sixth Sp1 motif, a Pax-4 motif, and a composite Sp1/MyoD element. The human and rat genes contain a binding sequence for Engrailed 1 (En-1) at a separate but close location upstream of the Sp1/GC boxes. The nearby upstream region contains also a HOXA3 binding sequence in human and an Aml-1 and C/EBP binding sequences in mouse (Fig. 2).

#### Potential regulatory regions outside the core promoter

The human, mouse, and rat *ADAM15 *genes were subjected to phylogenetic footprinting in order to localize potential *cis*-regulatory elements outside the core promoter. Sequence conservation analysis (Fig. 1C, upper panel, and data not shown) was complemented with a search for clusters of conserved TF recognition motifs, which would indicate locations of potential extra-core promoter regulatory elements (Fig. 1C, lower panel).

In all three species, the FLJ32785 intron 15 consists mostly of repetitive sequence with only short conserved elements lacking TF binding motif clusters in between (Fig. 1B, C). Consistently, reporter experiments showed that FLJ32785 intron 15 does not contain strong *ADAM15 *promoter activity-regulating elements.

The most prominent conserved non-coding *ADAM15 *regions were found in introns 1 and 2. Intron 1 region 3684 to 4228 and intron 2 region 4803 to 5244 are conserved across all three species and contain several conserved TF recognition motifs which are more tightly clustered in the intron 2 region (Fig. 1C). The conserved regions do not contain long open reading frames nor were corresponding transcribed sequences found from the GenBank, suggesting that the intronic conservation is not due to transcription of another gene.

Also the introns flanking the alternatively used exons contain obvious conserved regions. The low number of Match- and Consite-predicted TF binding sites suggests other potential role for these sequences from transcription regulation of *ADAM15 *or the downstream EFNA4. These regions might be conserved due their possible role in *ADAM15 *splicing or in the regulation of alternative splicing. Conservation in the introns flanking alternatively used exons has been reported also for other genes [25].

The human and mouse introns 3, 6, 10, 15, and 16 contain repetitive elements at corresponding locations, but only four of these 20 elements show conservation also in the type and sequence (Fig. 1B, mouse repeats not shown). Sine or Mir repetitive elements were not detected in the introns with long conserved regions or introns containing CpG-islands, as if their insertion would not have been tolerated.

### Three *ADAM15 *exons are used differentially in human tissues

**Figure 3:**
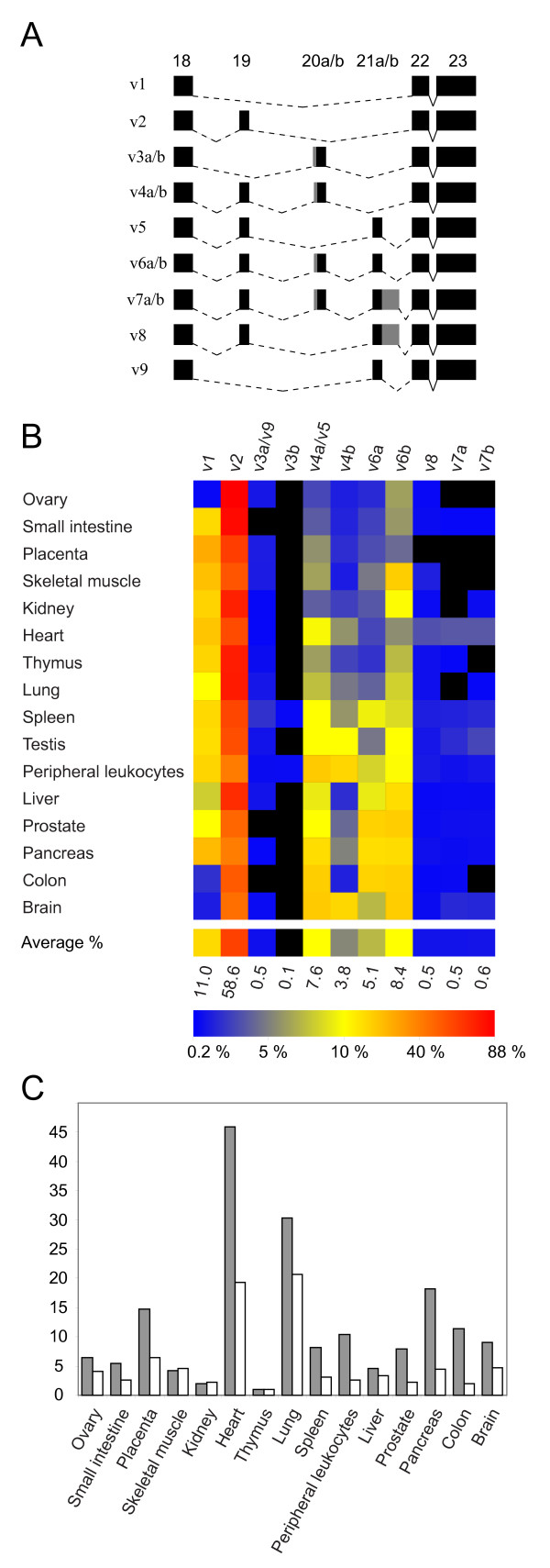
The alternative *ADAM15 *exon use in human tissues. (A) Schematic representation of the identified human ADAM15 mRNA variants. For clarity, only the cytosolic-encoding region containing the alternatively used exons is shown. The solid connecting lines indicate splicing of the constitutive exons and the dashed connecting lines indicated splicing of the alternatively used exons. The exons are shown as black boxes, with gray shading indicating the alternative 5'- and 3'-ends of the a/b variants of the exons 20 and 21, respectively. (B) *ADAM15 *mRNA variant profiles in normal human tissues, detected with fluorescence-PCR/capillary electrophoresis. The relative abundance of the mRNA variants (columns) within given tissue (rows) is indicated with the color coding. Black indicates relative abundance below 0.2% and colors from blue through yellow to bright red show the relative abundances from 0.2 to 88% (see the color key bar). The bottom row shows the average of the relative abundances in all examined tissues. (C) Real-time PCR (LightCycler) quantitation (solid bars) of *ADAM15 *expression level and that defined with fPCR/capillary electrophoresis as the combined peak area of all detected *ADAM15 *cDNA variants within given samples (open bars). The vertical axis shows the *TBP*-normalized expression levels relative to thymus (the lowest tissue expression, value set to 1.0). The similarity of the tissue distribution (Pearson correlation 0.902) determined with the two methods indicates that significant amounts of possible additional mRNA variants were not missed with the fPCR/capillary electrophoresis detection.

**Figure 4:**
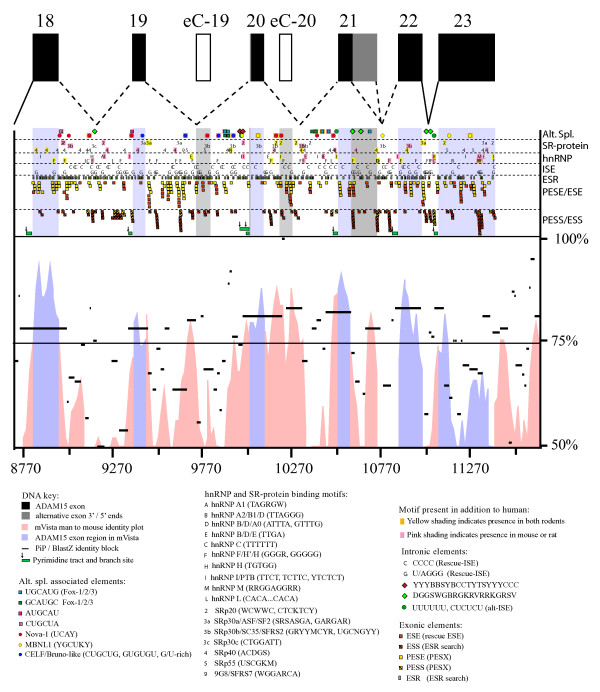
Splice-regulatory motifs in the alternatively spliced *ADAM15 *region. Predicted alternative splicing-regulatory protein recognition motifs within and flanking the alternatively used *ADAM15 *exons. The exon-intron structure of the human alternatively spliced ADAM15 region is shown at the top. The exons are shown as black boxes. The exon 21b-unique 3'-region is indicated with grey. The open boxes indicate the location of the putative cryptic exons eC-19 and -20. The dashed lines indicate the possible splicings of the alternatively used exons 19–21 and solid lines indicate the constitutive splicing. For simplicity, the lines do not depict the alternative use of exon-20 5'-splice sites (differing by only three nucleotides). The alternative splicing-associated sequence motifs are indicated by the symbols explained in the key shown below the figure. The different motif classes are shown separately in the sectors indicated on the right and explained in the key. The locations of putative branch sites are indicated with the small arrows on top of the pyrimidine tracts (green bars) under the splice-regulatory motif sectors. The human-mouse sequence-conservation is visualised with mVista and PiP/BlastZ under the splice-regulatory motif panel. For easier distinction, the exonic regions are shaded in the splice-regulatory motif and sequence identity panels. Also the nucleotide numbering relative to the translation start is shown under the panels

In addition to the previously reported human *ADAM15 *transcripts, nine novel mRNA variants were identified after the cloning and sequencing of over 100 reverse transcription-PCR (RT-PCR) products. The variants result from alternative use of the exons 19, 20, and 21 (Additional file 1, Figs. 1A, 3, 4). Two of these exons are alternatively spliced at one end, giving rise to the exon variants 20a/20b and 21a/21b (Figs. 1 and 4, Additional file 1). The exons 1–18, 22, and 23 were found in all examined *ADAM15 *transcripts, indicating that they are constitutively used. The variant 9 (Fig. 3) was not confirmed by sequencing but was indicated by the consistent appearance of the predicted size fluorescence PCR (fPCR) product and by RT-PCR amplification with a primer specific for the exon-exon junction 18/21 (data not shown). The sequences were submitted to GenBank [GenBank: AF314227, GenBank: AY560593–560601, GenBank: 560617].

#### ADAM15 mRNA variant profile in human tissues

Tissue distribution of ADAM15 mRNA variants was examined with fPCR of a cDNA panel representing 16 human tissues. Tissues showed distinctive *ADAM15 *variant patterns with 9 to 13 different variants at various levels (Fig. 3B). The average relative *ADAM15 *variant levels varied from less than 1% (variants 3b, 7a/b, 8) to 59% of the variant 2 (Fig. 3). Placenta showed the simplest and spleen and peripheral leukocytes the most complex *ADAM15 *variant profiles (Fig. 3B).

The exon 20a/20b usage ratio in human tissues, calculated from variants 6a/b, was on average 0.58 varying from 0.22 in skeletal muscle to 1.10 in spleen. This indicated non-constant ratio use for acceptors and that neither of the acceptor sites in the intron 19 NAGNAG was exclusively used in any examined tissue.

The *ADAM15 *mRNA levels in human tissues were investigated using two methods: by summing-up the mRNA variant levels determined in fPCR experiments and by the real-time quantitative PCR (qPCR) amplification of a fragment within the constantly expressed ectodomain-coding region. The tissue expression results paralleled each other (Pearson coefficient 0.902), corroborating the reliability of the *ADAM15 *mRNA variant profiling assay and indicating that the amplified fPCR fragments are parts of full-length *ADAM15 *transcripts. This also suggests that, in addition to the comparability of individual *ADAM15 *variant levels within given tissue, the variant levels could be comparable also between different tissues. An exception to this is the testicular expression for which no reliable housekeeping marker gene was available for normalization [26].

### Alternative splice-regulatory elements in the *ADAM15 *gene

Conservation of the alternative use of the *ADAM15 *exons 19 to 21 in man and mouse motivated the comparative examination of the human, mouse, and rat *ADAM15 *gene sequences for the identification of the sequence elements associated with the regulation of alternative splicing.

#### Splice and branch sites in the alternative introns

Roughly half of the information for splicing has been estimated to reside in splice and branch site sequences [27,28]. The 5'ss sequences in the introns 18 (AGgtatga), 20 (AGgtaaat), and 21a (AGgtaacg) deviate from the consensus donor sequence (AGgtragt) at +4/+6, +5, and +5 positions, respectively. Also, the calculated binding energies of U1 snRNA base-pairing with these 5'ss (-4.90, -6.20, and -5.10 kcal/mol introns 18, 20, and 21, respectively) are weaker than in average human 5'ss (-6.53 kcal/mol). However, all the 5'ss-scores in the introns flanking the alternative exons are in normal range (data not shown). Intron 18 3'ss (tcatgcatagGGCAG) ASD-workbench score (3.62) and intron 19 branch site score (2.65) are low while the other 3'ss scores (7.08–9.18) and branch/pyrimidine tract scores (3.35–3.85) in the alternatively used introns are normal.

The second half of the information for splicing regulation is located outside of the splice and branch sites, in the exon and intron sequences. Figure 4 shows the distribution of the potential splice-regulatory factor target motifs in the alternatively spliced region of human *ADAM15 *gene.

#### Exonic splice enhancer and silencer (ESE, ESS) sequences in alternatively used ADAM15 exons

Constitutive human exons typically contain three to seven Rescue-ESE clusters (average 5.2) [27]. The alternatively used human *ADAM15 *exons 19, 20a/b and 21a contain one/zero, one/zero, and three/six ESEs/putative exonic splice enhancers (PESE) found with the Rescue-ESE [27]/PESX [29] predictions, respectively. All ESEs and PESEs in exon 21a are in two clusters (Fig. 4). The number of ESE-finder-predicted SR-protein binding motifs in the alternative *ADAM15 *exons is one third lower than the average in the constitutive *ADAM15 *exons (data not shown). Furthermore, only three SR-protein binding motifs were localized to alternative exons (Fig. 4). Hence, the alternative *ADAM15 *exon splicing might be less enhanced by ESEs than the constitutive exons.

The alternative *ADAM15 *exons 19, 20a/b, and 21a lack ESSs/PESSs and binding motifs for heterogeneous nuclear ribonucleoproteins (hnRNP) altogether and contain lower than average number of exonic splice-regulatory sequences (ESRs). While 12.9 ESRs were localized to *ADAM15 *exons on average, the alternatively used exons 19, 20a/b, and 21a contain seven, eight, and two ESRs, respectively. Despite the low usage in *ADAM15 *transcripts, the unique 3' part of the exon 21b contains several ESE, PESE, ESS, and ESR motifs as well as SR-protein and hnRNP binding motifs (Fig. 4).

#### ESE and ESS sequences in two cryptic ADAM15 exons

The GenBank EST sequences indicate locations for two rarely used cryptic exons within introns 19 and 20, referred to as eC-19 and eC-20 (for cryptic exon) (Fig. 4). Both cryptic exons contain several exonic splicing enhancer and silencer motifs (ESEs/PESEs and ESSs/PESSs). Transcripts containing these exons were not detected with exon specific primers in RT-PCR or fPCR, suggesting that they are not functional exons.

#### ESSs, ESEs, hnRNP and SR-protein target motifs, and introninc splice enhancers (ISE) in the alternative ADAM15 introns

In contrast to the alternative exons, the introns flanking them were rich in ESEs and ESSs as well as in hnRNP and SR-protein binding motifs (Fig. 4). The intron 19 contains several hnRNP H/F binding motifs and the intron 20 especially many conserved SRp20, SRp30b, SRp40, hnRNP F/H, and hnRNP B/D/E binding motifs. Conserved hnRNP H/F binding motifs are present near the intron 21a ends. Furthermore, several G- and C-rich ISEs were scattered throughout the alternative introns. The number of the G- and C-rich ISEs parallel the high guanine and or cytosine content, respectively, in the introns 18–21.

#### Alternative splice regulation-associated motifs in the alternatively spliced ADAM15 region

The four most significantly alternative splicing-associated upstream sequence motifs (AUGCAU, CUGCUA, GCAUGC, and UGCAUG) predicted in Brudno *et al*. [30] were found in ADAM15 alternative introns (Fig. 4). Of the predicted motifs, GCAUGC and UGCAUG contain a recognition sequence (GCAUG) for Fox-family alternative splicing regulator proteins [31]. The intron 18 contains an AUGCAU and CUGCUA motifs close to the 3'- and 5'ss, respectively. 2/2, 2/2, and 0/1 GCAUGC/UGCAUG motifs are in central location in the introns 19, 20, and 21, respectively. In addition to the human *ADAM15*, six of the eleven corresponding motifs are present also in at least either one of the rodents.

Binding motifs for the neuronal splice-enhancer Nova-1/2 were located in the introns 18, 19, and 20 (four, one, and four, respectively). Five out of nine Nova-binding motifs were found in the corresponding location also in both rodents (Fig. 4). The exon 19 contains one and the intron 19 three binding motifs for the CELF/BrunoL family proteins. Corresponding intronic motifs were found also from mouse and/or rat. Muscleblind-like protein 1 (MBNL1) binding motifs were found in the exon 20 and in the introns 20 and 21. The exonic MBNL1 binding sequence is conserved in human, rat and mouse. Exon skipping-associated C-rich or G-rich motifs [32] were located in the introns 18, 19, and 21 (Fig. 4). The intron 18 G-rich motif is present also in both rodents and the first motif in the intron 21 is present also in mouse. Altogether 62% of the alternative splicing associated elements in the human *ADAM15 *exon 18 to intron 21 region were found also at the corresponding positions in mouse or rat, 34% being present in all three species.

### Deduced cytosolic ADAM15 isoform tails contain different sets of potential SH3-interaction motifs

**Figure 5:**
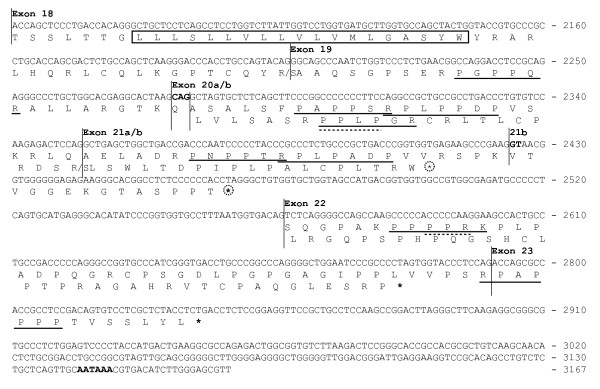
The cDNA sequence and amino acid translation of human *ADAM15 *alternatively spliced cytosolic tail-encoding region. The horizontal lines indicate the exon/intron boundaries. The a and b forms of the exon 20 differ by three nucleotides shown in bold. The adenylation signal is shown in bold. The start of the exon 21b-unique region is indicated with 21b. The upper translation corresponds to the mRNA variants containing the exon 19 and the lower translation corresponds to the alternative variants without the exon 19. The last amino acid encoded by the exons 18 and 19 depends on the next joined exon; both translations are shown separated by a slash. The transmembrane sequence is boxed. The putative SH3 domain-binding motifs (+XXPXXP and PXXPX+) in translations are underlined with solid line, and WW binding motifs (PPLP and PPR) with dashed line (P, proline; X, any amino acid; +, arginine or lysine). The putative NMD-targeting inframe stop codons are circled.

All characterized *ADAM15 *mRNA variants encode common ectodomain and transmembrane parts, and vary only at their cytosolic-encoding region. Variants encoding isoforms without the transmembrane part were not detected. Depending on the presence of the first alternative exon 19, *ADAM15 *mRNA variants are translated along either of the two alternative reading frames in the cytosolic-encoding region, downstream of exon 18 (Fig. 5, Additional file 1).

The variants 2, 4a/b, 5, 6a/b, 7a/b, and 8 with exon 19 (Fig. 3A) are translated along the first reading frame after exon 19 (Fig. 5). Since the first reading frame is open and the lengths of the alternative exons 20a, 20b, or 21a (72 nt, 75 nt, and 72 nt, respectively) are divisible by three, their inclusion does not change the reading frame and their alternative use changes the ADAM15 cytosolic tail in modular fashion. Therefore the deduced protein isoforms either contain or lack the segments encoded by these exons while the carboxyl end is invariable. The first reading frame contains an inframe stop codon in the exon 21b and thus the *ADAM15 *variants 7a/b and 8 (Fig. 3B) encode truncated tail isoforms (Fig. 5). The variants with the exon 19, translated along the first reading frame, contain up to seven PxxP-type Src-homology-3 (SH3)-binding motifs in five separate proline clusters in their cytosolic tails (Fig. 5). The proline clusters are encoded by the exons 19, 20a/b, 21a and the 22/23 junction (Fig. 5).

The *ADAM15 *variants lacking the exon 19 (1, 3a/b, and 9) are translated along the second reading frame downstream of the exon 18. Along this frame, exons 20a/b (variants 3a/b, Fig. 3B) encode also a proline cluster with SH3-binding motif (Fig. 5); ADAM15 isoforms (3a/b) containing this motif appear to show different SH3 protein interactions from those of isoforms translated along the first reading frame (our unpublished results). The variants 1 and 9 do not encode known SH3 interaction motifs.

The *ADAM15 *variants 7a/b, 8, and 9 are predisposed to nonsense mediated mRNA decay (NMD) pathway since they contain an inframe stop codon further than 55 nt upstream of exon-exon junctions 21b-22 and/or 22–23. This suggests that proteins encoded by these *ADAM15 *variants would not likely be produced at high level, consistent with their low mRNA levels (Fig. 3). The variants 1, 3a, and 3b contain an inframe stop codon at exon 22 only 16 nucleotides upstream of the last exon-exon junction and are thus not likely to be targeted to NMD.

### ADAM15 comprises a subgroup with ADAMs 8, 9, 12, 19, and 28

**Figure 6:**
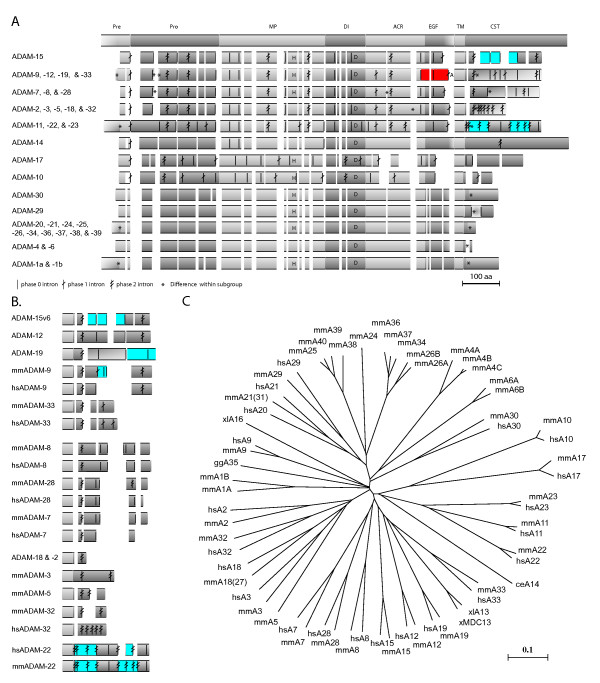
Schematic presentation of aligned ADAM proteins and gene structures. (A) Schematic presentation of aligned ADAM proteins arranged into 13 subgroups according to similarities in protein sequence and gene structure. The schematic structure at the top shows the ADAM domain structure: Pre, Pro, MP, DI, ACR, EGF, TM, and CT denote the pre-, pro-, metalloprotease, disintegrin, ADAM cysteine-rich, EGF-like, and transmembrane domains, and the cytosolic tail. The intron locations are shown by the vertical lines with none to two crossing tilted lines indicating the intron phase 0, 1, or 2, respectively. The alternatively used exons within the cytosolic-encoding region of *ADAM15 *are indicated with blue. The alternatively used exons 17 and 18 of *ADAM33 *and -*9*, respectively, are indicated with red. The alternative last exon used in the soluble isoform-encoding *ADAM12 *is indicated by the letter A. Regional differences in the number of amino acids within ADAM subgroup alignment are indicated by asterisks. The location of intact metalloprotease active sites and the disintegrin loop are indicated with letters H and D, respectively. (B) Cytosolic tail-encoding gene structures of ADAMs. Alternative exon use is indicated with blue. (C) Phylogenetic tree depicting the amino acid sequence-similarities between the ADAM proteins.

The ADAM15 exon-intron organization and protein sequence were compared to other human and mouse ADAMs and nematode ADAM14. Based on genomic and protein sequence similarities, ADAMs fell into 13 subfamilies. The *ADAM *exon-intron structures and a phylogenetic tree depicting the amino acid sequence similarities of the deduced ADAM proteins are shown in Figure 6.

The overall *ADAM15 *exon-intron structure is most similar to *ADAM*s 8, 9, 12, 19, and 28. The intron/exon structure of the extracellular (ectodomain) and transmembrane encoding regions in *ADAM15 *is highly similar to that in 12 *ADAM *gene family members (Fig. 6A). In contrast to the ectodomain, the structure of cytosolic-encoding gene region varies greatly between different ADAMs, and only five ADAMs show intron phases and locations partially conserved with *ADAM15 *in cytosolic-encoding region. The *ADAM*s 8, 9, 12, 19, and 28 share 3, 3, 4, 3, and 3 introns with conserved phase and location, respectively. Also at the protein sequence level, ADAM15 is most similar to ADAMs 8, 12, 13, 19, and 28. ADAM9 shows similarity with ADAM15 at cytosolic tail sequence (data not shown) as well as in the gene structure encoding it.

*ADAM15 *differs from other ADAM genes by three notable features. First, human and mouse *ADAM15 *lack an intron present in almost all other multiple-exon *ADAM*-genes (intron 14 of other *ADAM*s, Fig 6A). Another difference is the absence of a part of the exon 17 (Fig. 6A) which encodes a conserved membrane-proximal ADAM-specific protein motif, present in the majority of ADAMs from *C. elegans *to humans. This exon segment is absent also from ADAMs -7, -8, -28, -10, and -17. Third, ADAM15 is the only ADAM, in addition to ADAM22 [33], with conserved alternative splicing in cytosolic-encoding exons. In other ADAMs alternative use of cytosolic-encoding exons is restricted to either mouse or man (Fig. 6B).

## Discussion

This report describes characterization of the human *ADAM15 *gene and promoter, comparative analysis of potential transcriptional and alternative splicing-regulatory elements in human and rodent *ADAM15 *genes, and the first demonstration of complex differential alternative *ADAM15 *splicing in human tissues.

*ADAM15*, or its chromosomal neighborhood, appears to have been under selection pressure towards shorter length. *ADAM15 *gene is one third of the average human gene length [34,35] and the shortest of multiple-exon *ADAM *genes (some *ADAM *genes contain only one uninterrupted open reading frame). Although the number of *ADAM15 *exons is two and half times the human average and the exons are of normal size, the average *ADAM15 *intron length is less than one tenth of the human average [36]. The length of some *ADAM15 *introns is close to the theoretical minimum [37]. Moreover, *ADAM15 *appears to have lost an intron which is present in other ADAM genes.

The present results indicate several transcription start sites for human *ADAM15*, in contrast to the single start site reported for the mouse gene [20]. Their location suggested the promoter position within a CpG island proximal to the translation start, which was confirmed by the reporter expression experiments. The wide tissue distribution of *ADAM15 *expression is consistent with the CpG-rich promoter type [38]. Consistent with the presence of multiple transcription starts, the promoter lacks CAAT and TATA boxes, and contains a cluster of conserved Sp1/GC-box motifs.

While significant extra-promoter transcriptional regulatory elements were not found upstream of the *ADAM15 *promoter, the introns 1 and 2 contain highly conserved regions with clusters of conserved regulatory motifs. These could contribute to the transcriptional regulation since genomic sequence conservation is considered to indicate purifying selection and thus functional importance [39,40]. Alternatively, these regions could play a role in the splicing of the exons 2 or 3. Since the intronic splice-regulatory elements typically lie within 200 bp from exons [41], the central location of the conserved region in the intron 1 argues against a role in splicing whereas the conserved intron 2 region adjacent to exon 3 could be associated with its splicing. A third alternative explanation, that these regions were regulatory or coding parts of a gene in the complementary strand, is unlikely since no evidence was found for a complementary gene. The possible role of these conserved regions remains elusive.

The expression of *ADAM15 *appears to be remarkably regulated at the post-transcriptional level. For the first time, the present results demonstrate that normal human tissues display different complex patterns of at least 13 *ADAM15 *variants. Yamamoto and coworkers have earlier demonstrated the presence of *ADAM15 *mRNA variants corresponding to human v5 and v6 in mouse cell lines and some tissues [20,21]. The shortest *ADAM15 *variant (v1) has also been reported from cultured human cells [22]. Here, the fPCR method enabled identification of several novel variants as well as more precise and quantitative examination of the variant profiles. Otherwise the studies so far have considered the *ADAM15 *variant 2 as "the" *ADAM15 *and potential contribution of other variants has not been addressed.

All permutations of the alternative *ADAM15 *exons were not detected, suggesting that certain exon combinations are not generated or that they are unstable. The low levels of some variants might at least partially be due to their potential susceptibility to NMD, since their translation stop codon is located further than 55 nt upstream from an exon-exon junction [42]. Consistently, the variants 7a/b, 8, and 9, containing a potential NMD-targeting stop codon in the exon 21, were detected at very low levels. Variants lacking the exon 19 and containing the 21b, which would also contain a potential NMD-targeting stop codon, were not detected. In contrast, the stop codon in variants 1 and 3 is located only 16 nt upstream of the exon 22/23 junction and thus these variants should not be targeted to NMD. Correspondingly, the variant 1 was detected in several tissues at relatively high levels. The low level of variants 3a/b suggests that transcripts containing the exon 20a/b as the only alternatively used exon might not be efficiently generated.

Chance skipping of exons with weak splice signals could as such lead to complex splice patterns. However, the distinct *ADAM15 *splice variant patterns in cell lines and tissues indicate stringent regulation by specific factors. Alternative use of the corresponding exons in humans and rodents indicates at least some conservation of regulatory mechanisms, which should appear as conserved elements in the gene sequence. Hence, the human and rodent *ADAM15 *genes were comparatively examined for conserved alternative splicing-associated sequence motifs.

Splicing is primarily regulated through modulation of splice and branch site recognition and subsequent spliceosome assembly [43-45]. The binding strength of U1, U2AFs, and U2 snRNPs to their complementary sequences determines how easily the splice and branch sites will be skipped. This is further controlled by regulatory proteins which either facilitate or suppress the splice site selection depending on the type of the binding protein and the location of the binding site. The regulatory motifs are classified into exonic and intronic splice enhancers (ESE, ISE) and silencers (ESS, ISS) [46]. However, this nomenclature is not unambiguous; e.g., an ESS can enhance the splice site recognition if located within an intron [47]. Also the relative location within an exon or intron can influence the mode (enhancement/suppression) or potency of some regulatory elements [48].

The alternatively used *ADAM15 *exons appear to lack strong exon definition. Weak splice donor and branch sites, atypical 5'ss sequences, in particular a non-consensus nucleotide at +5 position, and low ESE numbers have been associated with alternative exon use [27,30,49-51]. Thus the weak 5' splice and branch sites, substitution of the +5 g in the 5'ss, and the scarcity of ESEs indicate that the alternative *ADAM15 *exons might be predisposed to skipping due to little exonic splice enhancement and inefficient splice site recognition. On the other hand, they are almost devoid of exonic suppression elements, i.e. ESS and hnRNP binding motifs. Hence, the alternative *ADAM15 *splicing is likely not controlled through exonic but rather through intronic motifs, consistently with the abundance of splice enhancer and repressor motifs in the flanking introns.

The available data do not allow prediction of the exact regulatory role of individual motifs but some suggestions can be made based on their location, type, and conservation. The hnRNPs have been shown to regulate alternative splicing through competitive pre-mRNA binding [44,45,52,53]. The conserved hnRNP I binding motifs in the intron-18 pyrimidine tract close to the U2AF site and the hnRNP H/F and B/D/A0 binding motifs close to the 5'ss in introns 21a and 21b, respectively, are potential splice-regulatory sites for which the hnRNP might compete. The hnRNPs have also been suggested to regulate alternative splicing through RNA loop-out mechanisms, in which homotypic interaction of pre-mRNA-bound hnRNPs enhances the use of splice sites by bringing them into proximity [54]. On the other hand, the model posits that if the looped-out RNA segment includes splice site(s), the corresponding exon would be skipped. The number and location of hnRNP H/F binding motifs in introns 19, 20, and 21 suggest that a looping-out mechanism may regulate splicing of the alternative *ADAM15 *exons 20 and 21.

The introns flanking the alternatively used *ADAM15 *exons contain several ESEs, SR-protein binding motifs, and ESSs possibly regulating the splice site use. The ESEs and SR-protein binding to motifs near the branch sites in the introns 19 and 20 may affect the downstream exon splicing via regulating branch site and 3'ss complex formation [44,55]. The SR-binding motifs just downstream of the exons 18, 19, and 21 could enhance their splicing by facilitating U2, U2AF, and U1 binding [56]. ESEs and SR-binding motifs in the unique region of exon 21b might enhance the distal 5'ss use. However, the ESSs in this region might enhance the selection of weak intron 21a 5'ss over the downstream 21b 5'ss. This could suppress the exon 21b use, as suggested by a recent study of the general role of intronic ESS motifs [47].

Fox-family proteins [57] are obvious candidate regulators of alternative *ADAM15 *splicing, suggested by the conserved Fox-binding motifs in the introns 19, 20, and 21. The Fox proteins have been shown to specifically affect the alternative splicing of their targets in several tissues [31,58-65]. Fox-binding elements have been shown to function as downstream splice enhancers or upstream silencers in mammals [65], and it remains to be elucidated which Fox-intron interactions might regulate the alternative splicing of individual *ADAM15 *exons.

The CUGCUA and AUGCAU motifs have also been associated with alternative splicing [30] although their cognate regulatory proteins have not been identified. Their location close to the 5'- and 3'ss in *ADAM15 *intron 18, and conservation of the CUGCUA between human and rat, suggest that these motifs might contribute to the regulation of alternative use of *ADAM15 *exon 19.

Other potential intronic regulatory elements in the alternatively spliced *ADAM15 *region are the conserved recognition motifs for splice factors of Nova, CELF, and MBNL families. These proteins regulate alternative splicing mostly as intronic upstream splice silencers and downstream enhancers [66-69]. However, their competitive binding with any splice factors could have an effect independently of the relative exon position. The uneven distribution of motif types in *ADAM15 *introns suggests that each of these splice factor families could affect the splicing of *ADAM15 *exons differently.

Alternative exon use might provide a novel physiological mechanism for functional ADAM15 regulation. Differential splicing of the three cytosolic-encoding exons in human tissues indicates differential regulation of *ADAM15 *variant production. This regulation appears to be disturbed in cancer cells which show aberrant *ADAM15 *variant patterns [19]. However, it remains to be investigated whether the *ADAM15 *mRNA variants are translated into protein isoforms in human tissues. Endogenous production of a longer ADAM15 protein isoform has been demonstrated in cultured mouse cells [20]. Although the molecular function of ADAM15 is poorly understood it has been associated with e.g. cell migration. In one cell type ADAM15 enhanced cell migration [70] whereas it inhibited motility in another [71], suggesting different function in different cells. It is intriguing to speculate that the differential splicing of *ADAM15 *transcripts could be a regulatory mechanism for such differences.

An obvious consequence of alternative *ADAM15 *splicing is the differential propensity of ADAM15 protein isoforms to regulatory cytosolic interactions. Thus alternative splicing could affect the cytosolic regulation of ADAM15 function or subcellular targeting. The cytosolic region of the deduced ADAM15 isoforms contains different sets of recognition motifs for signaling and adaptor protein interaction domains such as SH3. Several cytosolic protein tyrosine kinases, mitotic control factors, and cytoskeletal adaptors have been shown to interact with "the" ADAM15 (i.e., isoform-2) [3,72-74]. Yamamoto and coworkers have demonstrated that mouse ADAM15 isoform corresponding to human isoform-6 bound more strongly to Src family proteins than the "normal" form (isoform-2) and this interaction was enhanced by a phorbol ester in mouse cells, a treatment known to activate ADAM metalloproteases [20,21]. Our unpublished results indicate differential SH3-interactions for human ADAM-15 isoforms *in vitro*.

The intron/exon structure and deduced protein sequences indicate that ADAM15 comprises a subfamily with ADAMs 8, 9, 12, 19, and 28. The similarity suggests that the subfamily members may share functional and/or regulatory characteristics. *ADAM15 *is the only subfamily member for which the alternative use of the corresponding exons in the cytosolic-encoding region is conserved in between humans and mice. Another difference is the apparently lost intron from the ectodomain-encoding part of human and mouse *ADAM15 *genes. Its presence in almost all other ADAM genes indicates that this intron was deleted from an ancestral gene before the divergence of human and rodent lineages. The possible functional parallels with other subgroup members provide clues for further elucidation of the physiological and pathological roles and regulation of ADAM15.

## Conclusion

The human *ADAM15 *gene sequence, genomic structure, and promoter were characterized. For the first time, differential alternative *ADAM15 *splicing was demonstrated in normal human tissues, indicating that alternative exon use is a physiological post-transcriptional mechanism regulating ADAM15 expression. The genomic exon-intron structure is identical between human and rodent genes, and the considerable sequence-similarity of the human *ADAM15 *promoter with the corresponding region in mouse and rat, including several conserved transcription factor recognition motifs, suggests similarities in transcriptional regulatory mechanisms. Also, the alternative use of the same *ADAM15 *introns in human and mouse indicates conservation of at least some mechanisms regulating alternative splicing.

The present results provide important clues for further studies. Knowledge of the *ADAM15 *gene structure and transcriptional control elements are important prerequisites for better understanding of its mis-regulation in cancers and other diseases as well as for the elucidation of its physiological role. As alternative splicing is emerging as an important mechanism increasing the functional diversity of gene expression, characterization of the regulation mechanisms of alternative *ADAM15 *exon use will also provide novel information of alternative splicing in general. *ADAM15 *is particularly useful for monitoring changes in splice-regulation because the variant profiles arising from the alternative use of the three tandemly located exons can be readily examined with the straightforward and sensitive fPCR assay.

## Methods

### Cell lines

Human cell lines HCC-1954 (ATCC CRL-2338), MDA-361 (ATCC HTB-27), HCC-202 (ATCC CRL2316), and SK-BR-3 (ATCC HTB-30) were provided by the IMT Cancer Biology Laboratory (Institute of Medical Technology, University of Tampere) and cultured according to American Type Culture Collection (ATCC) recommendations [75].

### ADAM15 gene sequencing

PAC clone containing the *ADAM15 *gene sequence was identified from an arrayed human genomic PAC-library as described in [23]. PAC-DNA was purified with Qiagen Miniprep reagents (Qiagen, Hilden, Germany) and used as the template for PCR amplification of overlapping *ADAM15 *gene regions. Sequencing confirmed that the PAC-clone 240C8 contains the whole ADAM15 gene region. Over 100 PCR and sequencing primers were designed based on sequence information of human ADAM15 cDNA (GenBank accessions U46005 and U41767) and the unfinished human genomic sequence up- and downstream of the *ADAM15 *coding sequence (GenBank accession AL451085.6).

Amplified cDNA and gene fragments were gel-purified with MinElute gel extraction reagents (Qiagen) and directly sequenced or cloned. Sequencing was done with BigDye sequencing reagents according to the manufacturer's cycle sequencing protocol (Applied Biosystems, Foster City, CA, USA). Sequencing reactions were analysed with ABI Prism 310 and 3100 sequencers, and ABI data collection software, version 1.0.1 (Applied Biosystems). Genomic *ADAM15 *sequence contig was assembled and manually aligned with *ADAM15 *cDNA and EST sequences using the Genedoc sequence editor program [76,77]. The *ADAM15 *gene sequence was submitted to the GenBank [GenBank: AF314227].

### *ADAM15 *transcription start site determination

A successive PCR strategy was used for the transcription start site determination. Briefly, one downstream primer at a fixed position along the ADAM15 cDNA was used in PCR reactions against upstream primers, at successive positions upstream along the genomic sequence. Gene-specific cDNA template was reverse-transcribed with *ADAM15 *exon 5 antisense primer CCTGGTAGCAGCAGTTCTCC from 5 μg HCC-1954 and SK-BR-3 cell line total RNA with Superscript II reverse transcriptase (Invitrogen, Groningen, Netherlands). *ADAM15 *5'-target fragments were amplified with a common exon 2 antisense primer CTTCTCTGACTCTGCCTGCTGC paired with a series of sense primers (successively further up along the genomic sequence) that covered the putative *ADAM15 *promoter region and upstream region containing the last exons of the upstream-neighbour gene FLJ32785, at both transcribed and intronic positions. The upstream primers, with the genomic locations relative to the *ADAM15 *translation start ATG were as follows: (FLJe10/-5447) CTTCTGCCAGATCGATGACC, (FLJe15/-3338) GCGGATCCTGTTCCTCTACA, (FLJi15/-1419) AGCTCTCAGGGAGGGCTAAA, (FLJe16/-638) GTGTACTGCTGGTCGTGCTG, (FLJe16/-473) AAATGCCCTGTACGCTTCAC, (-355) GGAGGCCGCGAAGCTACTA, (-170) CTTTGCCGGGGGCGGAGCT, (e1/-159) GCGGAGCTTGGCTTGGGGC, (e1/-149) GCTTGGGGCCGGGTGGGA, (e1/-130) GGGGCGGGCCGGGGCGGGGC, (e1/-114) GGGCCTGGTGGCCGCGCG, (e1/-100) CGCGGCGCTGCTGGGTTCT, (e1/-86) GTTCTCCGAGGCGACCTG, and (e1/-1) CATGCGGCTGGCGCTGCTCT; (i and e indicate intronic and exonic hybridization sites of the primers, respectively).

PCR reactions were carried out for 30 and 35 cycles, in the presence of 8% DMSO, and the amplified DNAs were analysed with agarose gel electrophoresis. The products clearly detectable after 30 cycles of PCR were considered as normal *ADAM15 *expression, and the products that appeared only after 35 PCR cycles were judged as minor forms.

### *ADAM15 *cDNA variant cloning and luciferase reporter plasmid construction

Total RNA was isolated from the cultured cells using Trizol reagent with standard protocol (Invitrogen). 2 μg of total RNA from SK-BR-3, HCC-1954, MDA-361, and ZR-75-1 cells was reverse-transcribed using Superscript II reverse transcriptase and T_24_V-primer (V denotes any deoxynucleotide except T). The full-length *ADAM15 *cDNA was PCR-amplified using the primer pair GTTCTCCGAGGCGACCTG (sense e1/-86)/CGCAGAGTGTTGCTTGACAG (antisense e23/2598). Most of the cytosolic tail-encoding *ADAM15 *cDNAs were cloned as partial ADAM15 cDNAs with the upstream primers GGGCACAGGAATGTCGAAG (e15/1958) or GATGCTTGGTGCCAGCTACT (e18/2127) and the downstream primer CGCAGAGTGTTGCTTGACAG priming within the common 3'-untranslated region of the *ADAM15 *cDNA variants (e23/2598). The PCR products were gel purified and TA-cloned into topoisomerase-coupled pCRII or pCR2.1 plasmid vectors (Invitrogen). Over 100 clones containing cytosolic tail-encoding cDNA variants were sequenced.

For the promoter analysis constructs, six different sized *ADAM15 *upstream regions were inserted in forward orientation to the upstream cloning site of the enhancerless luciferase-reporter plasmid pGL2-Basic (Promega, Madison, WI, USA). Briefly, *ADAM15 *upstream region -3231 to -23 was amplified from the 240C8-PAC DNA with a common BamHI-overhang antisense primer AGTCGGGATCCCGAGGGCAGCAAGTGCGGAACAG and sense primers AGCAGAAGGCTCCGGTAAGT (-3231), XhoI-overhang CTGCCGCTCGAGCTGCAGGAACATGCTGGGATTCCTTGAC (-1582), and XhoI-overhang GAACCGCTCGAGCGGACTCCGCACAGTTGCGGGGAA (-266). The BamHI- and XhoI-primer restriction sites and the native sites XhoI, ApaI, and PstI were used in double digestion to produce cohesive ends for -3108/-23, -1582/-23, -266/-23, -1178/-23, and -415/-23 fragments. The restriction enzymes were purchased from Fermentas (Fermentas, Vilnius, Lithuania). The sixth *ADAM15 *construct (pGL2--266/-170+-121/-23) was a coincidental PCR-deletion-form found during the screen for -266/-23 promoter clones. All pGL2-*ADAM15 *constructs were sequenced to verify their insert sequences.

### Luciferase reporter assay

For each pGL2-*ADAM15*-construct, pGL2-Basic (control plasmid, luciferase without *ADAM15 *insert), and pGL2-Promoter (SV40 promoter), two triplicate sets of transfections were done with both SK-BR-3 and HCC-1954 cell lines. The cells were transfected with 4 μg pGL2-plasmid for each 3.2 cm well together with Lipofectamine Plus reagents (Invitrogen). The luciferase activity was measured with Promega luciferase assay according to the manufacturer's protocol (Promega, Madison, WI, USA) and 1254 Luminova luminometer (Bio-Orbit, Turku, Finland) or Luminoskan Ascent multi-plate luminometer (Thermo/Labsystems, Vantaa, Finland). The measured luciferase activities were normalized to the total protein determined with Detergent Compatible Protein Assay (Bio-Rad, Hercules, CA, USA) and Multiskan EX spectrometer (Thermo/Labsystems, Vantaa, Finland).

### Analysis of *ADAM15 *expression and mRNA variant profiles in human tissues

*ADAM15 *expression and alternative ADAM15 exon use in normal human tissues were studied with real-time qPCR and fPCR, respectively. The human tissue cDNAs (MTC multiple tissue panels I and II) were purchased from Clontech (Mountain View, CA, USA).

#### Real-time qPCR

The *ADAM15*-primer pair GAGAAAGCCCTCCTGGATG (forward/exon 10) and GGGCAGAATTCAGGCAAGT (reverse/exon 14) and the *TBP *(TATA box-binding protein)-primer pair GAATATAATCCCAAGCGGTTTG (forward) and ACTTCACATCACAGCTCCCC (reverse) were used in real-time qPCRs to examine the *TBP*-normalized *ADAM15 *expression level in the tissue samples. The reactions were done with QuantiTect SYBR Green PCR reagents (Qiagen) using LightCycler thermal cycler (Roche, Mannheim, Germany).

#### Fluorescence-PCR and fragment analysis

*ADAM15 *mRNA variants were detected from the human tissue cDNAs using PCR with the 5'-6-carboxyfluorescein (FAM)-labeled forward primer CTCAGCTCAAAGCAACCAGCTG (exon 17–18) and the reverse primer GGTCTGGAGGGTACCACTAGG (exon 22–23). Amplified DNAs and ROX-1000 DNA size standard (Applied Biosystems) were diluted into HiDi formamide (Applied Biosystems), and resolved with capillary electrophoresis and detected using ABI Prism 3130 Genetic Analyzer (Applied Biosystems). This sensitive and quantitative method will be described in more detail elsewhere (Ortiz RM, Kleino I, Isola J, Huovila A-PJ: Aberrant ADAM15 alternative exon use in human breast cancer, submitted). The fragment lengths and volumes were analyzed with Applied Biosystems Genoprofiler software.

### *ADAM15 *gene sequence analysis

Mouse and rat genomic and cDNA sequences corresponding to the human *ADAM15 *genomic region (-3231 to 11799) sequence were acquired from the GenBank using nucleotide BlastN search of the non-redundant and HTGS databases [78], and mega-BlastN for assembled genomes. The acquired mouse *adam15 *gene sequence was used in the overall phylogenetic footprinting analyses, and rat *adam15 *gene region sequence in the promoter and splice regulatory motif analyses as well as for guiding the genomic mouse/human alignment in ambiguous regions.

The genomic regions were examined for repetitive sequences with the RepeatMasker program [79] and the CpG islands were detected with the CpGPlot program [80,81].

The matrix searches for transcription factor binding sites were done with the public version of Match [82] using the vertebrate TF binding site matrices of Transfac 6.0 database [83]. The cut-offs were set for the matrix similarity to 0.9 and for the core similarity to 0.9. Putative composite elements [84,85] within the promoter region were searched with the Compel Pattern Search 1.0 program [86].

Phylogenetically conserved TF binding sites in the genomic sequences were searched using the Consite [87] with following settings: only vertebrate transcription factors with minimum specificity of 9 bits and transcription factor score threshold 84% were searched, conservation cut-off 50, window size 50. The *ADAM15 *ORF was excluded from the analysis

The alignment for the sequence identity visualization tool mVista [88] was done with the MAVID [89] and for the visualization tool PiP-maker [90] with the BlastZ. The alignment used in the Consite analysis was assembled manually from the initial alignment of the human, mouse, and rat genomic and cDNA sequences made with the ClustalW program [91]. The alignment was manually modified in the Genedoc program to correspond to the colinear blocks found with the Blast2 program [92]. The visual comparison of all genomic and cDNA sequences was used to guide optimal alignment of the poorly aligned regions. This alignment was later found to be very close to alignment produced with BlastZ which uses similar but fully automated alignment algorithm based on blast-program [93]. The manual alignment was used in the Consite analysis because some regions, e.g. the promoter region containing simple repeats, didn't align optimally with any of the tested automated alignment programs. The MAVID alignment was used in the mVista and rVista analyses and BlastZ alignment in the PiP-maker because these programs required the use of the accompanying alignment programs.

The branch sites were located and scored with Branch-Site Anlyzer program implementing the algorithm described in Kol et al. [51] and with ASD-workbench [94]. The 5'- and 3'-splice site binding energies and consensus scores were determined with the Splice-Site-Analyzer developed by the Ast lab [95] and with the ASD-workbench [94]. The consensus sequences used for the branch, 5'-, and 3'-splice sites were from [95,96].

Exonic splicing enhancer/silencer motifs were searched for with the Rescue-ESE server (ESEs) [27], the PESX-server (PESEs/PESSs) [29], the ESE-finder [97], and the ESR-search (ESRs and ESSs) [47,48]. The searched splice-regulatory motifs were collected from the literature: C-rich and G-rich ISEs [96]; GT-rich (alternative ISEs) [98]; hnRNP A1 [99,100]; hnRNP A2/B1 [45]; hnRNP B/D/A0 [101-105]; hnRNP C [45]; hnRNP D0 [104]; hnRNP H/F [44,45]; hnRNP H [106]; hnRNP I [44,45]; hnRNP L [45,107]; hnRNP M [45]; SRp20 [45]; SRp30a [45]; SRp30b [45]; Srp30c [108]; SRp40 [45]; SRp54 (AAGAAG) [109]; SRp55 [45]; 9G8 [45]; Tra2-B (GAAGAA)n [43]; UGCAUG, GCAUGC, AUGCAU, CUGCUA, [30]; Alt-ISEs UUUUUU, CUCUCU [30]; Fox-1/-2/-3 UGCAUG [31,110]; Fox-1 GCAUG [65]; Nova-1/-2 [111]; CELF/BrunoL family and (CUG)x8 [68,112,113]; MBNL1, (CCUG)n and (CUG)n [114,115]; exon skipping-associated C-rich or G-rich motifs [32]. All motifs in the articles were searched for, but only those that were found in *ADAM15 *gene sequence are shown in Figure 4.

### ADAM family comparison

For comparison of ADAM15 to other ADAM family members, the genomic and protein sequences of human, mouse, and *C. elegans *(ADAM14) ADAMs were retrieved from the GenBank. The protein sequences were aligned according to sequence-similarity. The correspondence of the intron locations and phases to the protein sequences was determined with the Wise 2 program [116] and checked manually. ADAMs were divided into subgroups based on the similarity of the genomic structure and protein sequence. A phylogenetic tree depicting the amino acid sequence-similarities of the ADAM proteins was built in the Clustal X and visualized with the Unrooted (Fig. 6C).

## Authors' contributions

IK carried out the cloning, sequencing, reporter expression, and PCR experiments, the sequence analyses, and initial drafting of the manuscript. RO participated in the set-up of the fPCR/capillary electrophoresis experiments, data analysis, and contributed to the manuscript. AH participated in the planning of the study and finalization of the manuscript. All authors read and approved the final manuscript.

## Supplementary Material

Additional file 1 Exon-intron organization of the human *ADAM15 *gene. The exon sequences are shown in upper case (the middle column) and the introns in lower case (the right and left columns). The location of the first and last nucleotides of the introns, relative to the translation start adenine, are indicated above the sequences. The intron lengths and phases are shown in the right columns. The first and last exon nucleotides are numbered according to their location in the coding DNA sequence (CDS). The amino acid translation is shown under the exon sequence. The non-consensus nucleotides around the splice sites are colored red (a purine (R) instead of a consensus pyrimidine (Y) or vice versa) and blue (no R/Y change). Click here for file
