# Supplementary material for: ADAM15 gene structure and differential alternative exon use in human tissues
Source: BMC Mol Biol. 2007 Oct 15;8:90. doi: 10.1186/1471-2199-8-90 (PMC2148059; doi:10.1186/1471-2199-8-90)
Supplement: Additional file 1 — Exon-intron organization of the human ADAM15 gene. The exon sequences are shown in upper case (the middle column) and the introns in lower case (the right and left columns). The location of the first and last nucleotides of the introns, relative to the translation start adenine, are indicated above the sequences. The intron lengths and phases are shown in the right columns. The first and last exon nucleotides are numbered according to their location in the coding DNA sequence (CDS). The amino acid translation is shown under the exon sequence. The non-consensus nucleotides around the splice sites are colored red (a purine (R) instead of a consensus pyrimidine (Y) or vice versa) and blue (no R/Y change). [file 1471-2199-8-90-S1.pdf]

Branch seq gene<br>Splice acceptor seq.	CDS location exon # CDS location<br>exon seq.<br>exons translation	Gene <intron length><br>Splice acceptor seq.<br>Intron phase
FLJ32785 exon 16<br>-471<br>.GCACCGTCCTTCCCG	fusion intron 1<br>-470 -455<br>GTTAATAAAATGCCCT	fusion intron 2<br>-454<br>gtacgcttcacg.
ADAM15	1 exon 1 79<br>ATGCGGCTGGCGCT.. GCTCCCAAATATAG<br>M R L A L L P N I G	80 <1205><br>gtgagtcctccgcct.<br>phase 1
.ccctcag... 1284<br>.tcctgggacctgcag	80 exon 2 186<br>GTGGCACTGAGGAG.. AAAAGGTGCTTCAG<br>G T E E K V L Q	1392 <637><br>gtgagctctcactcc.<br>phase 0
.aggtgac... 2028<br>.ctctggtgcccacag	187 exon 3 263<br>ACCAGTCTGCCTGA.. CTGCTACAGAATAG<br>T S L P E L L Q N R	2106 <416><br>gtaatagtgatggtg.<br>phase 2
.ttagcac... 2521<br>.ttcttcactccacag	264 exon 4 342<br>GGAGTTGGTCCCAG.. AGGGACACACTTTG<br>E L V P G G H T L	2601 <161><br>gtgagtcaggccctc.<br>phase 0
.cactaat... 2761<br>.gttcccatgctgcag	343 exon 5 419<br>GAGAACTGCTGCTA.. TGCTCTGGGCTCAG<br>E N C C Y C S G L R	2839 <87><br>gtatactgggcggat.<br>phase 2
.ctctgac... 2925<br>.tttctttttttcttag	420 exon 6 612<br>AGGCTTGGTGGTCC.. GCCACATTCGCCGG<br>G L V V L H I R R	3119 <1283><br>gtgaggatgaatggc.<br>phase 0
.gcctgat... 4401<br>.tcttgtccatagtag	613 exon 7 675<br>AGGCGGGATGTGGT.. CTGATCACTCGGAG<br>R R D V V D H S E	4465 <79><br>gtgagcctgctggcc.<br>phase 0
.cttcat... 4543<br>.tctcttggcctacag	676 exon 8 744<br>GCCCAGAAATACCG.. TCTTGCTGGACACA<br>A Q K Y R L L D T	4613 <79><br>gtgagtgctggacag.<br>phase 0
.ccctgac... 4691<br>.ccagctgtctttcag	745 exon 9 914<br>TTCTTCCGGCCCCT.. GCCCAGCTGGTGAC<br>F F R P L A Q L V T	4862 <136><br>gtaagggccccagac.<br>phase 2
.cctgaac... 4997<br>.ctctctgtcccacag	915 exon 10 999<br>TGGTACTTCATTCT.. GAGGTGTGAACATG<br>G T S F S G V N M	5083 <482><br>gtgagttatttccag.<br>phase 0
.gacctac... 5564<br>.cctccccaatgacag	1000 exon 11 1148<br>GACCACTCCACCAG.. GAGGCCTCCACAGA<br>D H S T S E A S T D	5714 <86><br>gtaagtagctgcagg.<br>phase 2
.ccccag... 5799<br>.tgccaccctccccag	1149 exon 12 1323<br>CTTCCTACCAGGCC.. GTGGCTTCCTGGAT<br>F L P G L G F L D	5975 <274><br>gtgagcccctttccc.<br>phase 0
.ctctgat... 6248<br>.tgctcctgcccacag	1324 exon 13 1416<br>GACTGCGTCGATCC.. GTCAAAATTGCCAG<br>D C V D P Q N C Q	6342 <121><br>gtgggtagagactag.<br>phase 0
.cactgat... 6462<br>.atccaccctccacag	1417 exon 14 1721<br>CTGCGCCCGTCTGG.. TCCTGCACCCCTAG<br>L R P S G S C T P R	6768 <90><br>gtaagtgaggaaacc.<br>phase 2
.tactaac... 6857<br>.tccccctccccacag	1722 exon 15 1917<br>AGATGCCATTTGTG.. GTGGCCCTGGCCTG<br>D A I C G G P G L	7054 <268><br>gtgagcagcctgggt.<br>phase 0
.atctgat... 7321<br>.ctctccctgggtcag	1918 exon 16 1995<br>GTGTGTATAGACCA.. GCCATGGACATGGG<br>V C I D H H G H G	7400 <1114><br>gtgagctgggatggg.<br>phase 0
.tcctcat... 8513<br>.ccttgtacctcctag	1996 exon 17 2068<br>GTCTGTGACAGCAA.. CACTCAGCTCAAAG<br>V C D S N T Q L K A	8587 <230><br>gtagcatgggggtgg.<br>phase 1
.cccttaa... 8816<br>.gacttccgcccacag	2069 exon 18 2207<br>CAACCAGCTCCCTG.. ACCTGCCAGTACAG<br>T S S L T C Q Y R	8956 <419><br>gtatgagcatcacct.<br>phase 2
.gtctgag... 9374<br>.tttcctcatgcatag	2208 exon 19 2277<br>GGCAGCCCAATCTG.. CACGAGGCACTAAG<br>A A Q S G R G T K	9445 <582><br>gtgagtcccggatgc.<br>phase 0
.tgcctat...10026<br>.ctcctgccctctcag	2278 exon 20a/b 2352<br>cagGCTAGTGCTCT.. CCAAGAGACTCCAG<br>Q A S A L K R L Q<br>R L V L S P R D S R	10102 <86/414><br>gtaaatctgggccag.<br>phase 0
.ctctcac...10515<br>.cctctctctgttcag	2353 exon 21a 2424<br>GCTGAGCTGGCTGA.. TGAGAAGCCCGAAG<br>A E L A D R S P K<br>L S W L T *	10588 <269><br>gtaacggtgggggga.<br>phase 0
	21b 2566<br>TTTAATGGTGACAG<br>-	10730 <127><br>gtttgtttgcagaca.<br>phase -
.cccccac...10856<br>.ccttctctccctcag	2425 exon 22 2549<br>TCTCAGGGGCCAGC.. GTGGTACCCTCCAG<br>S Q G P A V V P S R<br>P L R G Q -	10982 <90><br>gtaggaggagccctg.<br>phase 2
.ctctgac...11071<br>.cttcccgcaatccag	2550 exon 23 2865<br>ACCAGCGCCACCGC.. TTGCAATAAACGTGACATCTTGGGAGCGTT<br>P A P P P -	
